# A dynamic and collaborative database for morphogeometric information of trilobites

**DOI:** 10.1038/s41597-023-02724-9

**Published:** 2023-11-29

**Authors:** Fernanda Serra, Diego Balseiro, Claude Monnet, Enrique Randolfe, Arnaud Bignon, Juan J. Rustán, Valentin Bault, Diego F. Muñoz, N. Emilio Vaccari, Malena Martinetto, Catherine Crônier, Beatriz G. Waisfeld

**Affiliations:** 1 Universidad Nacional de Córdoba, Facultad de Ciencias Exactas Físicas y Naturales, Córdoba, Argentina; 2 CICTERRA (Centro de Investigaciones en Ciencias de la Tierra), CONICET, Córdoba, Argentina; 3 Univ. Lille, CNRS, UMR 8198 – Evo-Eco-Paleo, F-59000 Lille, France; 4 IIMYC (Instituto de Investigaciones Marinas y Costeras), CONICET - Universidad Nacional de Mar del Plata, Mar del Plata, Argentina; 5 IGCyC (Instituto de Geología de Costas y del Cuaternario), Universidad Nacional de Mar del Plata - CIC PBA, Mar del Plata, Argentina

**Keywords:** Palaeontology, Palaeoecology

## Abstract

Modern morphometric-based approaches provide valuable metrics to quantify and understand macroevolutionary and macroecological patterns and processes. Here we describe TriloMorph, an openly accessible database for morpho-geometric information of trilobites, together with a landmark acquisition protocol. In addition to morphological traits, the database contains contextual data on chronostratigraphic age, geographic location, taxonomic information and lithology of landmarked specimens. In this first version, the dataset has broad taxonomic and temporal coverage and comprises more than 55% of all trilobite genera and 85% of families recorded in the Paleobiology Database through the Devonian. We provide a release of geometric morphometric data of 277 specimens linked to published references. Additionally, we established a Github repository for constant input of morphometric data by multiple contributors and present R functions that help with data retrieval and analysis. This is the first attempt of an online, dynamic and collaborative morphometric repository. By bringing this information into a single open database we enhance the possibility of performing global palaeobiological research, providing a major complement to current occurrence-based databases.

## Background & Summary

Understanding biotic responses to large scale environmental changes -either past or recent- is one of the main goals of macroecology and macroevolution. Major biological events that took place during the Palaeozoic, such as the Cambrian Explosion, the Great Ordovician Biodiversification Event (GOBE), and the mid-Palaeozoic Marine Revolution, shaped the marine life for the rest of the Phanerozoic and paved the way for the forthcoming configuration of current marine ecosystems^1–4^. Among the iconic organisms of this Era, trilobites are a fundamental group for the study of Palaeozoic benthic faunas and provide excellent opportunities for the understanding of mechanisms underlying ecological and evolutionary patterns at different spatial and temporal scales^5–9^. Several palaeontological studies have highlighted the critical role of trilobites in the signature of the Cambrian–Ordovician ecosystems and, although declined significantly during the Late Ordovician extinction, they continued being an emblematic group of the benthic ecosystems during the middle Palaeozoic^10^.

Palaeobiodiversity studies have classically examined taxonomic changes on the basis of the fossil record^11–15^. Indeed, understanding the patterns of diversity collapses and recoveries provides valuable insights into drivers of changes and helps to inform conservation activities in modern marine ecosystems in light of anthropogenic climate change^16,17^. Most of these studies are facilitated by the Paleobiology Database^18^ (PBDB) or the Geobiodiversity Database^19^ (GBDB), which are comprehensive, international collaborative, taxonomic and occurrence databases already available and widely used to assess macroevolutionary patterns and processes in deep time and for global to regional analyses^20–24^. Related studies, therefore, focused mainly on the taxonomic richness component of biodiversity. However, it is well-known that biological diversity is multifaceted (e.g.^25^), and morphological diversity is another key aspect to understanding ecological-evolutionary patterns in deep time^26^.

For extinct organisms such as trilobites, morphological disparity is highly relevant to understand and quantify macroevolutionary and macroecological patterns and processes (e.g.^5,27–30^). Modern morphometric-based approaches, such as landmarks and morphospaces, are also used as valuable metrics to quantify impacts and responses of marine ecosystems to large-scale changes (such as global warming) in terms of morphology and functional needs through deep time (e.g.^31–37^). Regarding morphological information, only small datasets linked to specific publications exist, while very few large scale morphological datasets are currently available (e.g.^38–40^). Regarding trilobites, there has been an increase in taxonomically comprehensive morphological databases^41^, such as outline data describing the shape of the cranidium^31^, semilandmarks describing the shape of the cephalon^42^, landmark and semilandmarks configuration on cephalic outline, glabellar and eye ridges^43^, landmarks and semilandmark data on cephala and pygidia^44^, as well as trilobite moult morphometric measurements^45^. Here, we develop a database for morphological information of trilobites that is also quantified by means of geometric morphometrics state-of-the-art methods. These approaches allow objective quantification of shape of organisms, ordinate major shape differences onto morphological spaces, and then quantify the occupation of this morphospace by various disparity metrics and compare them to phylogenetic patterns or known abiotic and biotic changes. The novelty behind TriloMorph is its dynamic and collaborative nature, thus promoting inclusive and sustainable work between researchers, approaching an open science framework.

Trilobites are extinct marine mostly benthic, mobile organisms forming the class Trilobita, one of the earliest known groups of arthropods. They first appear in the fossil record around 521 Ma ago and flourished throughout the lower Palaeozoic^46,47^, before slipping into a long decline, with all trilobite orders except the Proetida dying out during the Late Devonian^48^. The last trilobites finally disappeared at the end-Permian mass extinction about 252 Ma ago^49^. Trilobites were among the most successful of all early animals, thriving in oceans for almost 270 million years, with over 20,000 species having been described^41,50^. Due to their excellent fossil record and high diversity and abundance, they are an ideal group for analysing biotic changes during the Palaeozoic.

**Fig. 1:**
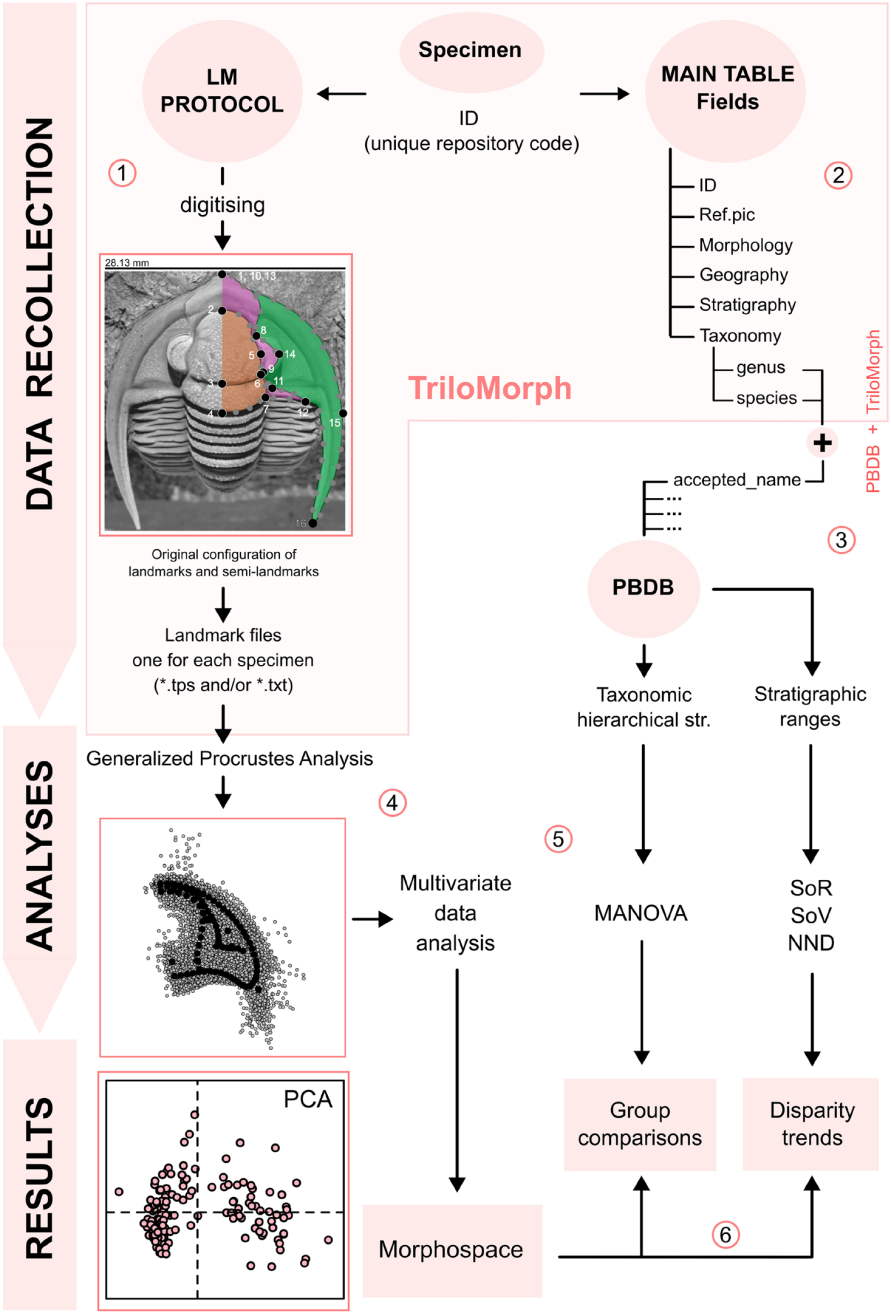
Flow diagram illustrating the main TriloMorph workflow: (1) LM protocol: the acquisition of morphological data of a specimen with unique repository code (ID) is carried out following the provided landmark protocol; after digitising, one landmark file is generated for each specimen. (2) Main table: specimen ID conforms the basic unit of entry in the main table that contains specimen-level traits and contextual characteristics (‘data.csv’). (3) PBDB + TriloMorph: taxonomic hierarchical structure and stratigraphic ranges can be obtained by merging TriloMorph data with occurrence and higher taxonomic information from the PBDB by using the accepted name from PBDB and species or genus names from TriloMorph, (4) Generalised Procrustes analysis: standardisation of landmark data. (5) Analyses: a variety of analytical tools can be used to quantify shape variation (e.g. multivariate data analysis, MANOVA, disparity measures) depending on the research goal. (6) Results: with this database and protocol it is possible to construct a morphospace to visualise patterns of shape variation in trilobites, carry out group comparative analyses or study disparity trends. A step by step explanation of the procedure and R function utilisation is given for the highlighted part (pink box) and an example on the analysis and results sections using Devonian genera from TriloMorph is provided in the R code TriloMorph-workflow. Abbreviations: LM: landmark; Taxonomic Hierarchical str.: Taxonomic Hierarchical structure; SoR: sum of ranges; SoV: sum of variances; NND: Nearest neighbour distance; PCA: principal component analysis.

In this first release of the openly accessible “TriloMorph” Database, we describe the general landmarking protocol and database functionality (Fig. 1). The initial dataset is focused on Devonian trilobites, and late Cambrian–Early Ordovician ones to a lesser extent, which have a very rich fossil record at times where major transitions of life happened^10,51^. The morphology of most Devonian trilobite genera with records in the PBDB has been digitized and compiled in the database which, in addition to these morphological traits, contains contextual data on chronostratigraphic age, geographic location, taxonomic information, and lithology of the landmarked specimens. TriloMorph is the first attempt of such an open online morphometric repository of extinct marine organisms that promotes and brings together data generated from the collaborative efforts of contributors in a dynamic manner. The possibility of integrating morphometric data with data from the established Paleobiology Database, which is the biggest online resource of fossil occurrence data, provides the opportunity to address large-scale palaeobiological studies.

## Methods

**Fig. 2:**
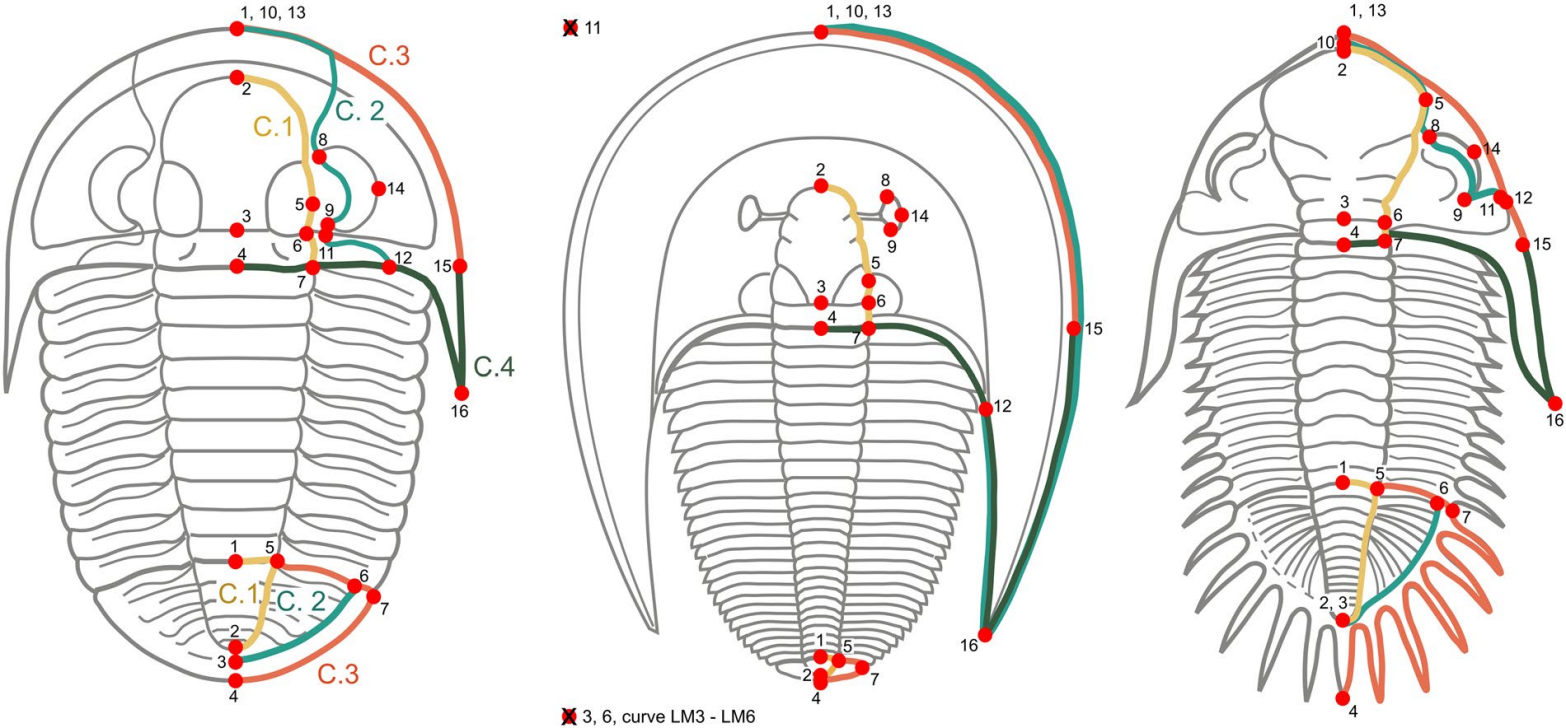
Template of landmarks and semilandmarks curves used in the TriloMorph database illustrated on three different genera (*Cyphoproetus*, *Harpes*, and *Kayserops*, from the left to the right). For *Harpes*, absent landmarks are referred to as crosses besides the figure. Figures adapted from Gon, S.M. III^114^, used with permission.

**Fig. 3:**
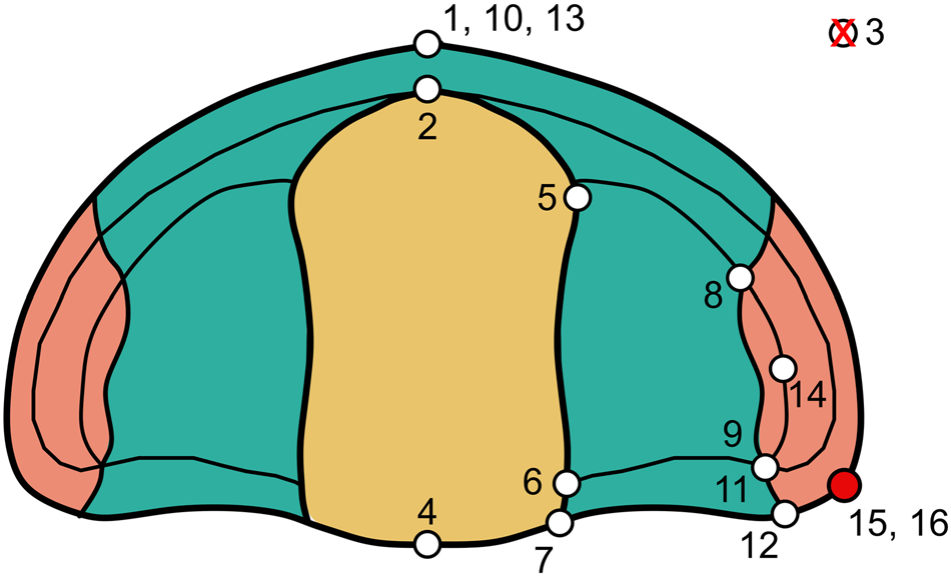
Schematic diagram of the cephalon of *Ellipsocephalus*, showing the alternative position of LM 15 in the template used in the geometric morphometric analysis. Figure adapted from Gon, S.M. III^114^, used with permission.

Morphological data were compiled from a landmark-based geometric morphometric approach^52^ to investigate the morphology of two major anatomical structures of trilobites, namely the cephalon and the pygidium. Specimens selected for digitization were compiled from the literature and belong to public collections with a unique repository code. Specimens were named after this alphanumeric identifier and digitised from their original 2D published pictures (Fig. 1, Data recollection). In this sense, 2D landmark configuration has proven to be adequate for capturing shape change in trilobites, despite the differences that may exist in the convexity of certain structures^53^. Landmarks and semilandmark curves were referred to the right half of the structures analysed, on a dorsal view of each specimen. If a specimen had the left side better preserved, the picture was mirrored prior to landmarking. When missing, a graphic scale was placed on each specimen prior to digitization. Morphology of the cephalon is described by 16 landmarks and 4 semilandmark curves, and the pygidium by 7 landmarks and 3 semilandmark curves (Tables 1, 2, Fig. 2). Geometric morphometrics enables describing the shape of organisms by specifying landmarks, which are homologous topological points identifiable in all studied specimens, as well as semilandmarks, which are equally-spaced points capturing the shape of boundary curves and of surfaces^54^. Semilandmark curves for the cephala represent the shape of the glabella, cranidial and cephalic outlines and the posterior part of the cephalon (Figs. 2, 3). Regarding the pygidium, the semilandmark curves represent the pygidial and axis outlines and the border furrow.

**Table 1:** Definition of the landmarks and semilandmark curves for the cephalon.

Landmarks	
Number	Definition
LM1	Anteriormost point of the sagittal cephalic length without spine
LM2	Anteriormost point of the sagittal glabellar length
LM3	Intersection between the sagittal axis and the occipital furrow
LM4	Posteriormost point of the sagittal cephalic length
LM5	Maximum transversal glabellar width
LM6	Intersection between the occipital and axial furrows
LM7	Intersection between the posterior margin and the axial furrow
LM8	Anteriormost end of the eye
LM9	Posteriormost end of the eye
LM10	Anterior facial suture at the sagittal line
LM11	Intersection between the posterior branch of facial suture and the posterior or lateral border furrow
LM12	Intersection between the posterior branch of facial suture and the posterior or lateral margin
LM13	Anteriormost point of the sagittal (or ex-sagittal) cephalic length (if spiny, LM13 at the tip of the spine)
LM14	Lateralmost external point of the eye
LM15	Cephalic width at the level of the posterior margin of the occipital ring (LM4). If that point cannot be located, it is defined as the extreme of the genal angle (see Fig. 3).
LM16	Tip of the genal angle or spine
**Semilandmarks (curves)**	
**Curve**	**Starting landmark - ending landmark**
Glabella (C.1)	LM2 - LM7
Facial suture (C.2)	LM10 - LM12
Anterior margin (C.3)	LM13 - LM15
Posterior margin (C.4)	LM4 - LM15

**Table 2:** Definition of the landmarks and semilandmark curves for the pygidium.

Landmarks	
Number	Definition
LM1	Anteriormost point of the pygidial axis
LM2	Posteriormost point of the pygidial axis
LM3	Border furrow at the sagittal point
LM4	Posteriormost point of pygidium at the sagittal line (if spiny LM 4 at the tip of the spine).
LM5	Intersection between the axial furrow and the anterior pygidial margin
LM6	Intersection between the anterior pygidial margin and the border furrow
LM7	Intersection between the anterior and the lateral pygidial margin
**Semilandmarks (curves)**	
**Curve**	**Starting landmark - ending landmark**
Axis (C.1)	LM1 - LM2
Border furrow (C.2)	LM3 - LM6
Margin (C.3)	LM4 - LM5

Landmark quantification may vary between specimens depending either on the preservation of the specimen or because the specific trait is not present in the respective taxon; for example eyes in blind taxa or specimens with no articulated cephala. In the case of an incomplete specimen selected for digitization, for example, a cephalon with a broken genal spine, we completed its morphology by taking in reference another specimen from the same publication. In exceptional cases where certain taxa do not have well preserved structures, drawings were used for digitization if they were based on a specific specimen with a repository code. However, it is frequent that some taxa lack certain structures; in these cases, we suggest removing these traits from the analysis. In the static release, 82% of the specimens in the database allow complete landmark acquisition in the cephala and 100% in the pygidia. Also, this first release includes only adults (early holaspid and holaspid) specimens.

Landmark and semilandmark selection will ultimately depend on the scope of the analysis. Thereby, here we present a comprehensive digitization protocol.

Several softwares exist for 2D landmark acquisition, such as ‘ImageJ’^55^, ‘tps’ series^56^, or the digitize2d() function in the ‘geomorph’ R package^57,58^. Here, we used both the ‘digitizeImages()’ function in the ‘StereoMorph’ package^59^ for R^60^, and the classical ‘tpsDig’ software^56^.

## Data Records

**Fig. 4:**
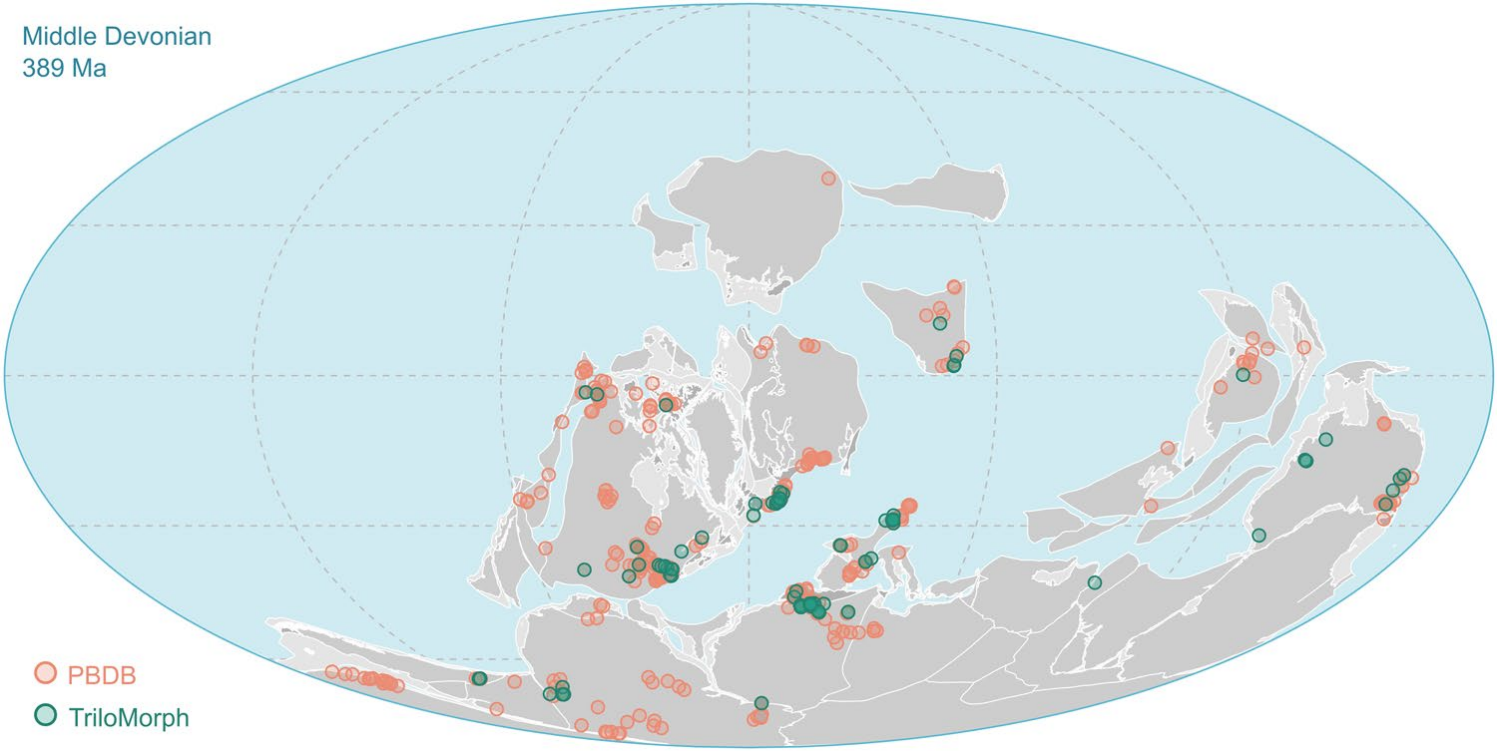
Palaeogeographic map of the Eifelian Stage (Middle Devonian) indicating the geographic location of the studied collections of trilobite specimens included in the TriloMorph database (green) and location of collections from the PBDB (pink) in order to show the geographic coverage of TriloMorph. Palaeogeography is reconstructed using the PALEOMAP model^115^ in GPlates^116^.

**Fig. 5:**
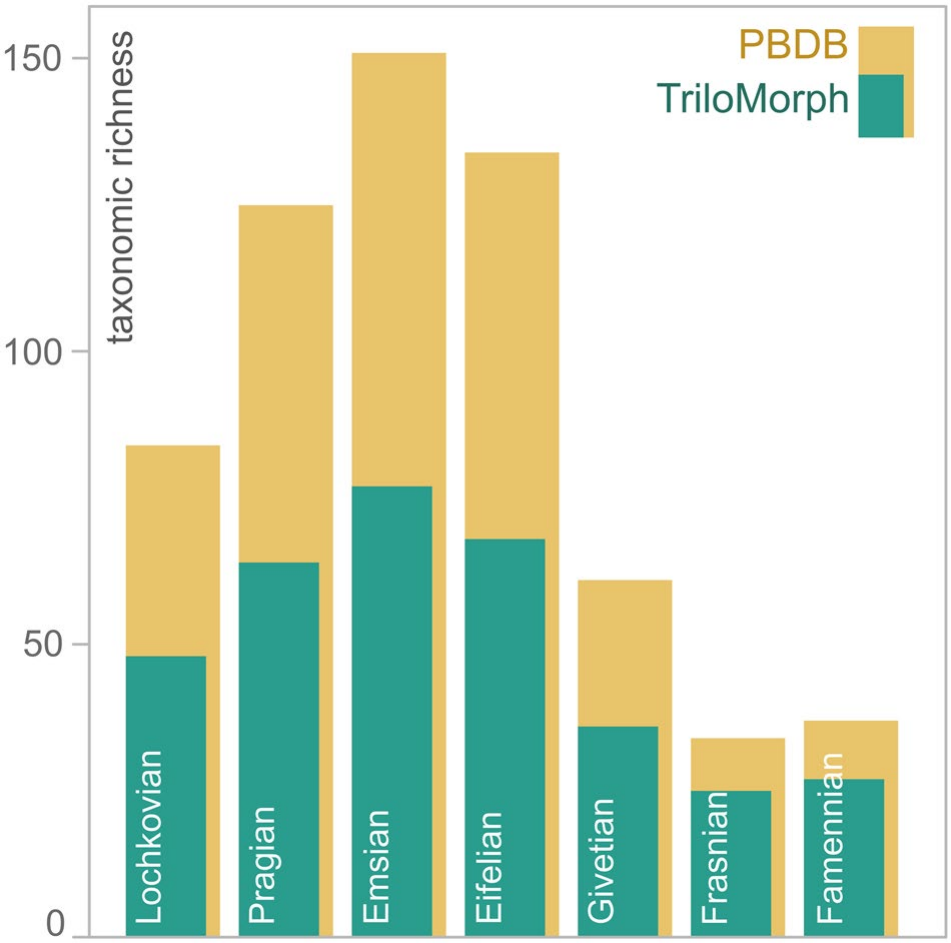
Amount of genera documented in the TriloMorph database (green) in relation to the number of trilobite genera for each Devonian stage recorded in the PBDB (yellow). Taxonomic richness was range standardised based on the Devonian occurrences recorded in the PBDB.

Here we provide the release of a geometric morphometric dataset of 277 specimens. The associated metadata has information down to specimen level, thus allowing, for example, to include several specimens of the same species for intra-specific analyses. Because it is a specimen-based dataset, TriloMorph allows users to carry out analyses at any desired taxonomic resolution. All specimens included in this release are linked to published references (i.e. peer-reviewed papers, taxonomic monographs, books etc., see references in the GitHub repository) and are derived from localities all around the world (Fig. 4). As a first step, and as a strategy to maximise taxonomic coverage of the dataset, we include one specimen per genus. The current version of the dataset has broad taxonomic and temporal coverage and comprises more than 55% of all trilobite genera and 85% of families in the PBDB in the Devonian geological stages (Fig. 5) and even some taxa yet lacking occurrences in the PBDB. Static releases of the database are available directly from the Universidad Nacional de Córdoba data repository^61^ and Zenodo^62^, while a dynamic collaborative version is available in GitHub (https://github.com/balsedie/trilomorph). The purpose of the TriloMorph GitHub repository is to allow the constant input of new data into the database. We also provide a step by step explanation of the procedure to upload new data and describe the R functions needed to download and analyse data hosted in this repository. Static releases are in the form of a compressed folder containing the following files and folders:

### Metadata

YAML file containing the contextual information of considered specimens (see below and Table 3). YAML is a human-friendly data serialisation language for all programming languages^63^.

**Table 3:** Definition of the fields describing the specimen-based table of contextual information (‘data.csv’) (*mandatory fields for subsequent classical analyses).

Field group	Field name	Description
	*ID	Unique specimen collection number (either the official repository number, or, if not available, a combination of the publication information illustrating the specimen (author_year_plate_figure))
	*ref.pic	Reference of the digitised specimen image
	*enter.metadata	person who entered the metadata (format: Surname initials)
	*enter.landmark	person who digitised images (format: Surname initials)
	comments	space to comment on any of the previous fields
Taxonomy	*taxonomy.orig genus	Original genus assignment in the publication
Taxonomy	*taxonomy.genus	Valid genus name of the specimen
Taxonomy	taxonomy.gen status	Open nomenclature qualifier for genus identification (such as ‘aff’, ‘cf’, or ‘?’, among others)
Taxonomy	taxonomy.gen_author	Author of the genus (surname(s) and year)
Taxonomy	taxonomy.subgen	Valid subgenus name
Taxonomy	taxonomy.orig sp	Original species assignment in the publication
Taxonomy	*taxonomy.sp	Valid species name (use binomial nomenclature)
Taxonomy	taxonomy.sp_author	Author of the species (surname(s) and year)
Taxonomy	taxonomy.subsp	Valid subspecies name
Morphology	*morphology.cephalon	Boolean value to inform about the digitised structure (‘TRUE’ if it corresponds to a cephalon, otherwise ‘FALSE’)
Morphology	*morphology.cranidium	Boolean value to inform about the digitised structure (‘TRUE’ if it corresponds to a cranidium, otherwise ‘FALSE’)
Morphology	*morphology.pygidium	Boolean value to inform about the digitised structure (‘TRUE’ if it corresponds to a pygidium, otherwise ‘FALSE’)
Morphology	*morphology.eyes	Boolean value to inform if the species has eyes (TRUE) or not (FALSE)
Morphology	*morphology.ontogeny	inform the ontogenetic stage of the specimen (early Holaspis, Holaspis, Meraspis)
Geography	*geography.locality	name of the locality from which the specimen was collected
Geography	geography.country	country of origin of the specimen
Geography	*geography.lat	latitude of the locality of origin of the specimen. Format: decimal coordinates
Geography	*geography.long	longitude of the locality of origin of the specimen. Format: decimal coordinates
Geography	geography.state	state of origin of the specimen
Geography	geography.county	county of origin of the specimen
Stratigraphy	stratigraphy.formation	name of the formation from which the specimen comes from
Stratigraphy	*stratigraphy.min_age	minimum age for the occurrence of the specimen (stage)
Stratigraphy	*stratigraphy.max_age	maximum age for the occurrence of the specimen
Stratigraphy	stratigraphy.ref_age	reference for age determination (format: Surname year, Surname Surname year, Surname *et al*. year)
Stratigraphy	stratigraphy.basin	name of the basin from which the specimen comes from Stratigraphy

### References.csv

A CSV-formatted file containing the bibliographic information of data sources. Each entry of the specimen-based table (‘data.csv’) contains the identification number of the data source, whose bibliographic details are provided in this ‘reference.csv’ table, which can then be used for citation purposes and should be credited in subsequent publications using the database. Contributors are also acknowledged in the accompanying file ‘contributors.csv’.

### Images

A folder containing the digitised images. Image files are named after the specimen identification number (i.e., collection/repository number, also present in the main table ‘data.csv’), and are appended with a suffix to identify the corresponding anatomic structure of the trilobite (“_C” and “_P” for the cephalon and the pygidium, respectively). File names should not contain spaces. All pictures have a graphical scale. Available formats are JPG and PNG. Images of cephala and pygidia are saved in different subfolders.

### Landmarks

A folder containing the shape data (landmarks and semilandmark curves) for each specimen separately. Similarly to images, landmark files are named after the repository code of the corresponding specimen appended with the suffix of the considered morphological structure (see above). Shape files created with StereoMorph (*.txt) or tpsDig (*.tps) are available. Shape files of cephala and pygidia are saved in different subfolders.

The database is, therefore, a collection of data files overseen by a main table designed to contain specimen-level traits for considered taxa. The basic unit of entry in this main table is that of a specimen, normally stored in public collections and with a unique alphanumeric identifier (**id**), that is also accompanied by contextual characteristics such as the publication describing this specimen (**ref.pic**), taxonomic information (**taxonomy.genus, taxonomy.orig_genus, taxonomy.gen_status, taxonomy.gen_author, taxonomy.subgen,** **taxonomy.sp, taxonomy.orig_sp, taxonomy.sp_author,** **taxonomy.subsp**), relevant morphological information (**morphology.cephalon,** **morphology.cranidium,** **morphology.pygidium,** **morphology.eyes,** **morphology.ontogeny**), geographic context (**geography.lat,** **geography.long,** **geography.country,** **geography.state,** **geography.county**), and stratigraphic information (**stratigraphy.formation,** **stratigraphy.min_age,** **stratigraphy.max_age,** **stratigraphy.ref_age,** **stratigraphy.basin,** **stratigraphy.environment**). These metadata are crucial for subsequent analyses of morphological disparity in a spatial, temporal and environmental context. Details about each field are described in Table 3. Although it is not the current scope of the database, the specimen-based structure of the database allows including several specimens of the same species to also perform intraspecific analyses.

In addition to the database, we also provide several R functions to read both StereoMorph and TPS data simultaneously (function ‘shapRead’ in the file ‘trilomorph-funs.R’) into a list structure, then to check homogeneity (number of landmarks, number of curves and constituting semilandmarks, presence of a scale, presence of missing landmarks) of the loaded landmark data, next to resample each curve to the same number of semilandmarks as requested by the user (function ‘shapFix’ in the file ‘trilomorph-funs.R’) and to finally provide a standard 3D landmark array (landmark values by landmark dimension and by specimen, see^64^). In case of inconsistencies among the selected specimens, the latter function automatically removes these specimens with missing landmarks.

## Technical Validation

The acquisition of landmarks for this database used softwares, such as tps^56^ and StereoMorph^59^, which have been abundantly used in palaeontology and biology for decades and have proven to be efficient and accurate, therefore, ensuring technical rigour (e.g.^52,64^).

The landmark template defined here is the result of a collaborative work among many trilobite specialists in our group (e.g^44,48,51,65–86^) and is also based on a long historical research of landmark-based protocols in trilobites (e.g^42,84,87–95^). It is designed to maximise trilobite shape description across all major trilobite orders.

**Fig. 6:**
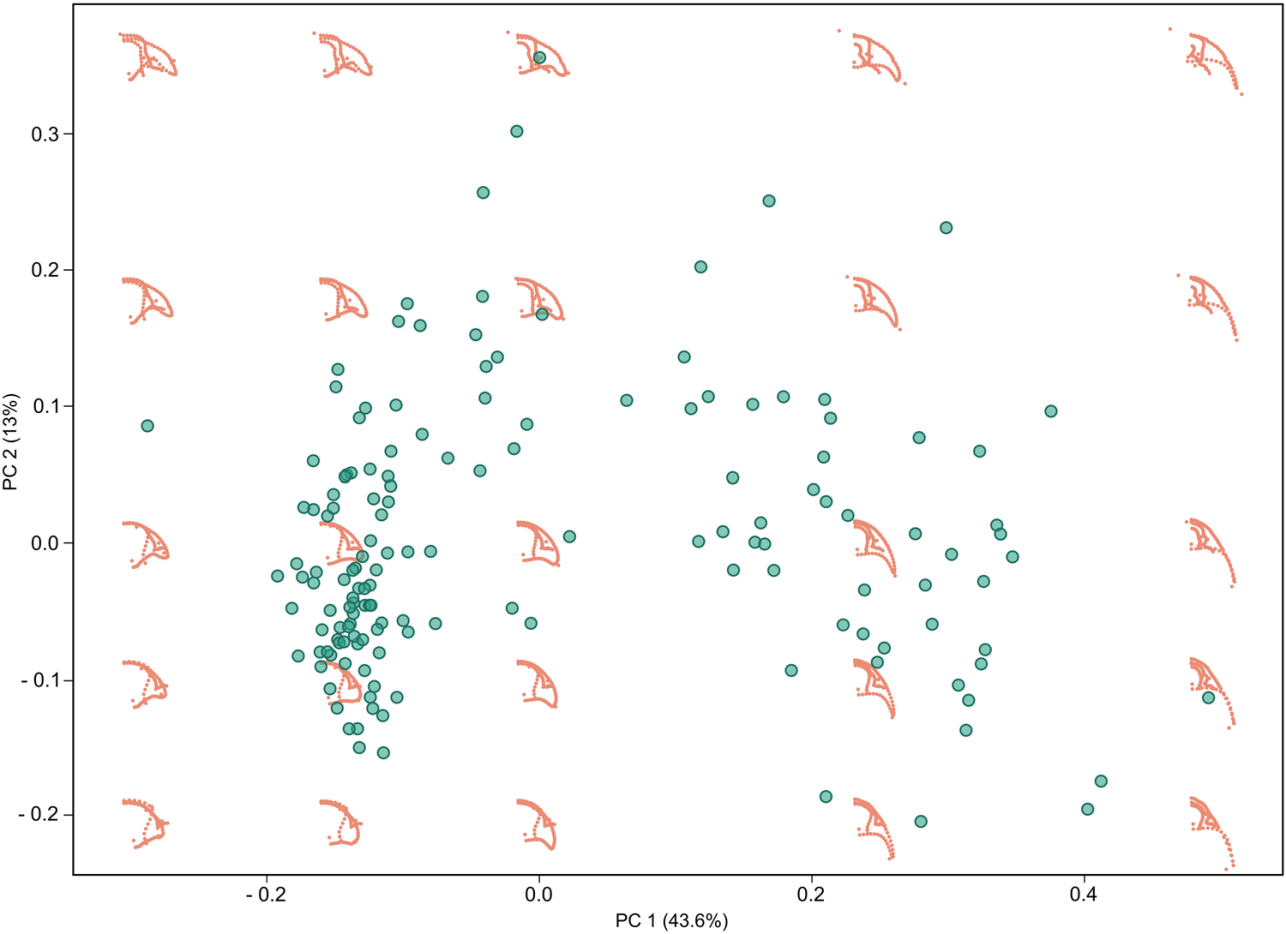
Trilobite morphospace using Devonian data from TriloMorph, virtual shapes are plotted in order to show intuitively how shape varies across morphospace. Note that our landmarking protocol is particularly sensitive to the main morphological changes described in the literature^96^. For example, the lower right quadrant represents mostly morphological diversity among Phacopidae, towards the upper part mostly Homalonotidae, Styginidae, Calmonidae, towards the lower left quadrant morphological diversity resembles Acastidae, Odontopleuridae, Aulacopleuridae, Proetidae.

One important concern might be the capability of the proposed landmarking protocol to recognize trilobite morphological variation across its whole -or at least most of the- phylogeny but also recognize variability within taxonomic groups. In this sense, our results of the trilobite morphospace indicates that the current protocol is particularly sensitive to the main morphological changes that have been previously described in the literature^96,97^ (Fig. 6). For example, our method is able to capture the cephalon outline, but also suture and glabellar outline and eye morphology, as well as key pygidial features, all of which have been interpreted as important traits when describing the morphological variability among trilobites^97^. Results using this protocol demonstrated its ability to distinguish main morphological variations across all orders and families present during the Devonian (see Usage notes), as well as at regional and local scales during the lower Ordovician^77^, and within a single family (Phacopidae) along its whole evolutionary history^44^.

However, there are some taxonomic groups that represent a challenge for the application of this protocol. For example, in several trilobite clades (e.g. some representatives of the Suborder Illaenina, and the Order Asaphida) effacement of cephalic and pygidial furrows is a common feature, making it difficult to determine certain landmarks and curves. On the other hand, there are several types of facial sutures, and therefore the curves and landmarks involved have been resolved to encompass these different patterns. For instance, for marginal sutures that run along the cephalic margins, LM 10 (anterior facial suture at the sagittal line) is placed together with LM 1 and so the cranidial outline is adjacent to the cephalic margin (e.g. harpetids); for sutures running parallel to the anterior border, LM 10 is positioned between LM1 and LM2 so the anterior part of the cranidial outline runs parallel to the margin (e.g. dalmanitids) (Fig. 2).

**Fig. 7:**
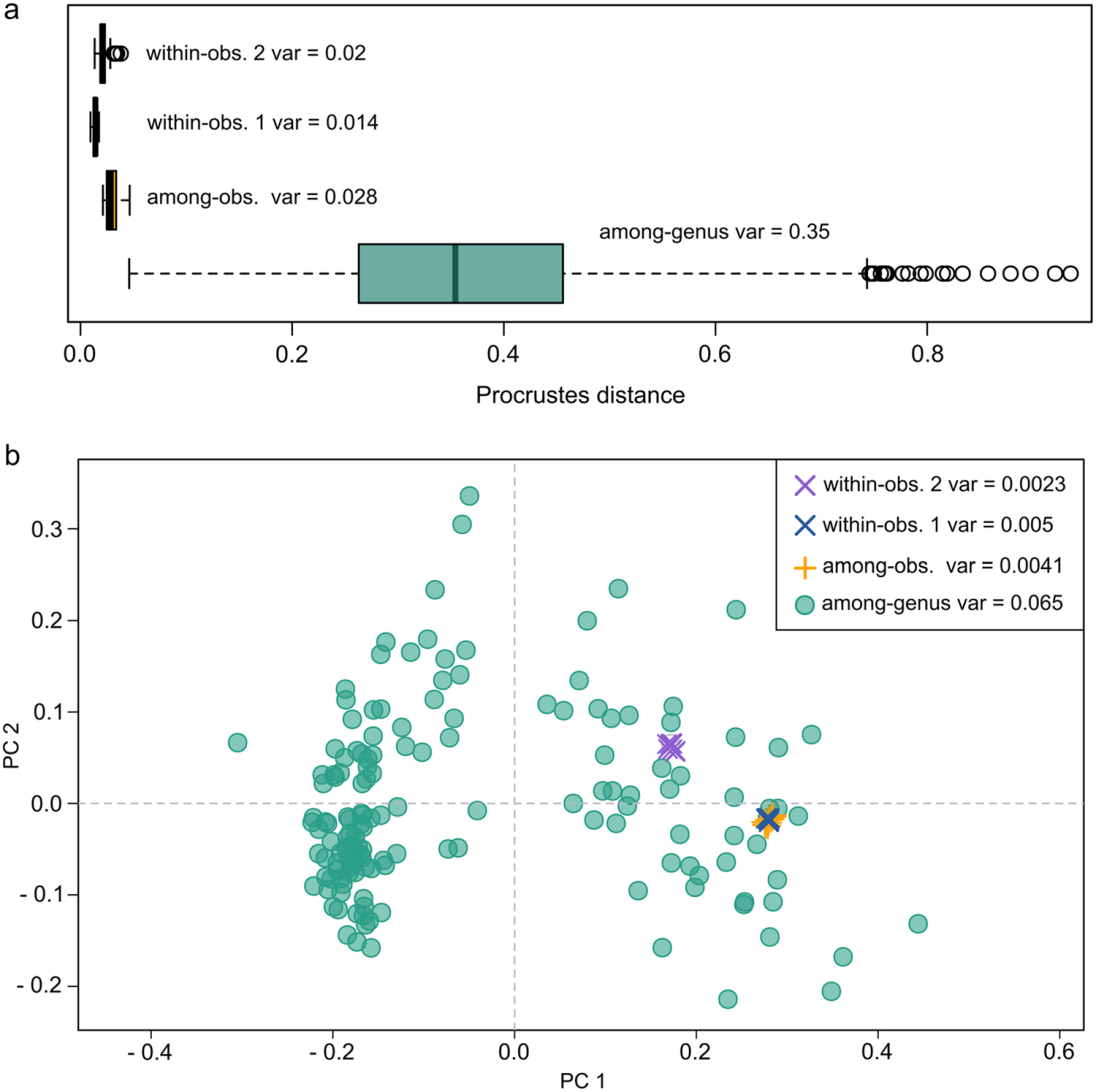
Morphological variability among specimens from two sources of error for morphogeometric data: within-observer (within-obs. 1, within-obs. 2), and among-observers (among-obs,), compared to among-genus variability. (**a**) Distribution of pairwise Procrustes distances among specimens within each group. (**b**) Morphospace for measurement error for specimens within each group.

Because the database is based on landmarked data obtained by different contributors and digitising software, we tested for variation on landmarked data as a measurement of data quality. We tested three different sources of error for morphogeometric data, namely within and between observer variability in the landmarking process, and variability between different landmark acquisition software. For this, we compared inter-generic variability in our dataset with variability in a single specimen based on (1) ten landmark and semilandmark configurations obtained by the same observer (within observer variability, two test cases) and (2) landmark and semilandmark configurations obtained by 8 different observers using StereoMorph and one observer using TPS (among observer variability). Morphological variability was estimated as (1) the pairwise Procrustes distances among specimens within each group: among-genus, among-observers and within-observers and (2) total multivariate dispersion in the morphospace for each group. Results indicate that within observer variability, i.e. strict measurement error (0.014 and 0.02), and among observer variability (0.028), which in turn includes different landmark acquisition software variability, are substantially smaller (one order of magnitude) than inter-generic variation (0.35), see Fig. 7.

A Kruskal-Wallis rank test confirms that the differences in pairwise Procrustes distances are statistically significant (χ² = 349.85, df = 3, p < 2.2e-16). The test for multivariate dispersion^98^ also indicates statistically significant differences (df = 3, F = 80.524, p < 2.2e-16). We further used a Tukey test to recognise which groups were significantly different based on their multivariate dispersions. Table 4 indicates that significant differences are present only when comparing inter-generic variability to among-observers and within-observer variabilities (within-obs.1 and within-obs.2). While among observer variability is higher than personal error, it is still non-significant (Table 4). It is worth mentioning that measurement error tests using TPS were carried out in a previous study (see supplementary material in^44^), which also indicated a negligible variation compared to inter – genus variation.

**Table 4:** Tukey test performed to identify significant differences between 3 sources of error for morphogeometric data: within-observer 1 and 2 (within-obs.1, within-obs.2), and among-observers (among-obs), compared to and among-genus variability. Asterisk (*) denotes significance.

Groups	difference	lower	upper	p
among-obs. - among-genus	−0.230	−0.298	−0.162	0*
within-ob.1 - among-genus	−0.236	−0.297	−0.175	0*
within-ob.2 - among-genus	−0.232	−0.294	−0.171	0*
within-ob.1 - among-obs	−0.006	−0.095	0.083	0.998
within-ob.2 - among-obs	−0.002	−0.092	0.087	0.999
within-ob.2 - within-ob.1	0.004	−0.080	0.088	0.999

**Fig. 8:**
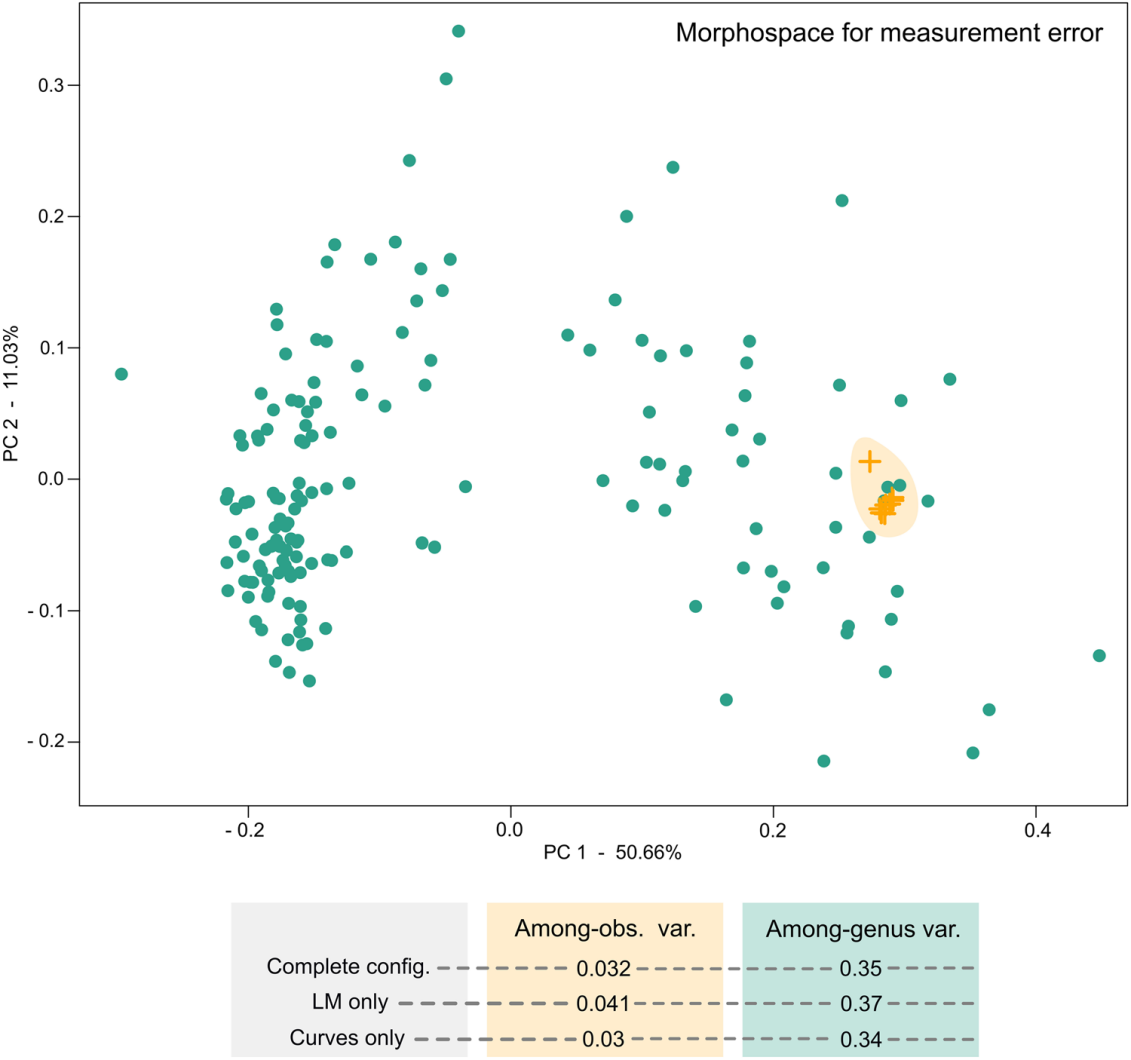
Morphological variability among specimens considering among-genus and among-observers sources of error. Note the increase in morphological variability among-observers when slightly changing the location of a landmark that makes up for the ending point of a semilandmark curve (orange shade). Abbreviations: config.: configuration, LM: landmarks; obs.: observer, var.: variability.

In a second analysis, to further assess the robustness of morphometric quantification, we tested variation on landmark data by intentionally misplacing a landmark. For this, LM 15 was slightly moved from its original position by one of the observers. Among-observers variability was tested considering both landmark and semilandmark configuration and only landmarks (excluding semilandmark curves). The former, increased as expected (0.032), although it remains non-significant with respect to inter-generic variation. However, variability considering only landmark configuration was higher (0.041), highlighting the importance of the location of landmarks that make up for the ending points of semilandmark curves (Fig. 8). Thus special consideration must be taken in the positioning of these landmarks.

These results indicate that at the current scale of the dataset, sources of error in the morphogeometric data are much smaller than the natural variability of the data. Therefore our protocol for obtaining data digitised by different contributors is reliable for large scale analyses. Our results are further supported by previous analysis using the same landmarking protocol^48^ where measurement error was lower than within species variability and significantly lower than among genera variability for a single morphologically conservative family (i.e. Phacopidae)^44^.

Finally, part of the database has already been used to investigate trends of morphological disparity of a specific group of Devonian trilobites^44^ and ecological dynamics of late Cambrian–early Ordovician trilobite assemblages^77^, therefore showing the applicability of our database and the usage notes discussed below.

The database is hosted on github^62^ and will be maintained on the long-term by DB, FS and other members of the database.

## Usage Notes

A database for morphological information of trilobites is presented herein. The database is open-access with the possibility to download information of interest and/or contribute to the dataset. Researchers who use the database are asked to cite this publication; we strongly encourage users to acknowledge main contributors to the analysed dataset. This novel database is ideal for quantitative analyses regarding morphological diversity, providing an excellent opportunity to explore macroevolutionary and macroecological dynamics.

**Fig. 9:**
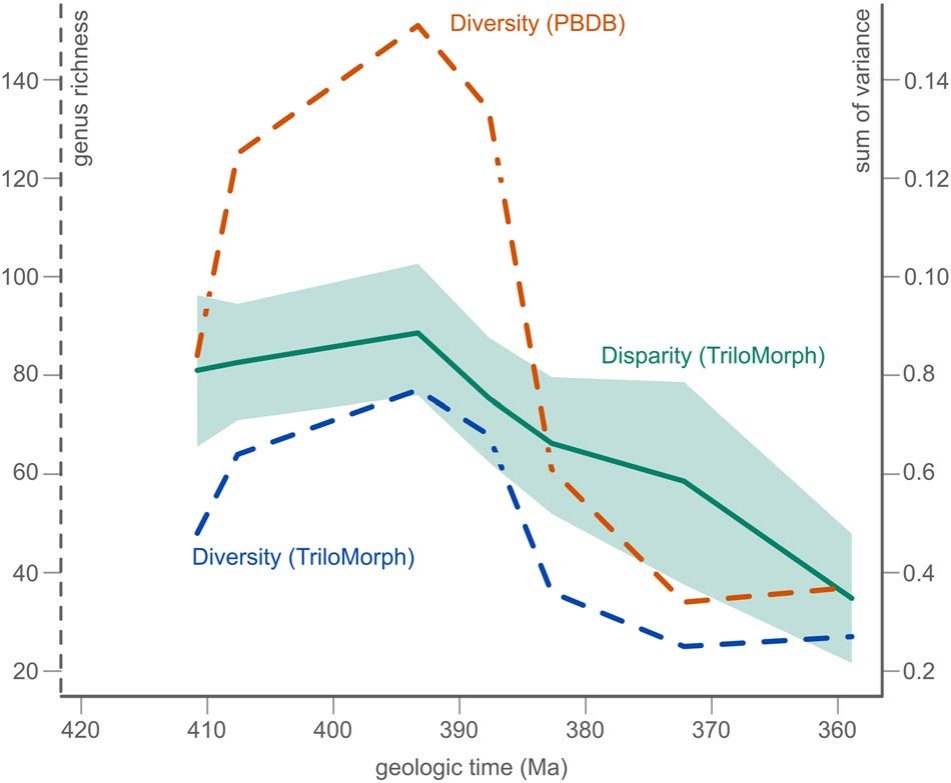
Devonian disparity and diversity trends based on TriloMorph morphometric data (measured as the SoV) and on trilobite genera present in the PBDB and in TriloMorph respectively.

Nowadays, geometric morphometric analyses are routinely used and there exist several softwares with various capabilities and options to perform such analyses (e.g. MorphoJ^99^, Morpheus^100^, PAST^101^), as well as several packages (e.g. geomorph^54^, shapes^102^, Momocs^103^) for R^60^. As an example, here we show the potential of the database for analysing morphological disparity through time, focusing on the Devonian. For this we follow a classical protocol schematized in Fig. 1 (the corresponding R code is available in the file ‘trilomorph-workflow.R’ provided with the TriloMorph database):

1. Load and match landmark data to the user-specified template, number of landmarks, and number of semilandmarks for each open curve (e.g. with the function provided with the TriloMorph database). The number of semilandmarks to be placed along the curves will ultimately depend on the user’s desired resolution of the shape data. In this contribution we resampled the 4 curves on the cephalon to 12, 20, 20 and 20 semilandmarks respectively because it was sufficient to fit our scope. Here, we use shape files created with StereoMorph (*.txt) or tpsDig (*.tps), but any other format can be used as long as it can be transformed into the standard array (‘landmark values by dimension by specimen’; see^64^). The function shapFix can be used to easily change the desired number of semilandmarks for each curve as an argument, and also will warn the user and automatically remove specimens with landmark data not fitting the desired template in order to continue with the general analysis.

2. Landmark data are standardised (superimposition step) by applying a generalised Procrustes analysis (GPA; e.g. the function ‘gpagen’ in the geomorph package), which facilitates the comparison of configurations by eliminating variation associated with differences in their location, orientation and size^52,104–107^.

3. Superimposed data are ordinated into a morphological space by applying a covariance-based principal component analysis (PCA; e.g. the function ‘plotTangentSpace’ in the geomorph package) in order to quantify and visualise patterns of shape variation. Actual shapes and/or virtual shapes can be plotted over this morphospace to illustrate and highlight major shape variations (Fig. 6).

4. Last, to quantify the morphospace occupation and its changes through time, we calculated the sum of variances (SoV; Fig. 9), which provides a measure of dispersion around the centroid of the group. Noteworthy, several other disparity indices have been developed to capture different aspects of morphospace occupation, and thus consideration of multiple indices is necessary to fully characterise changes in disparity^108–110^. These additional indices can be computed with the dispRity package^111^. In addition, taxonomic richness was standardised according to the Devonian occurrence records from the PBDB.

**Fig. 10:**
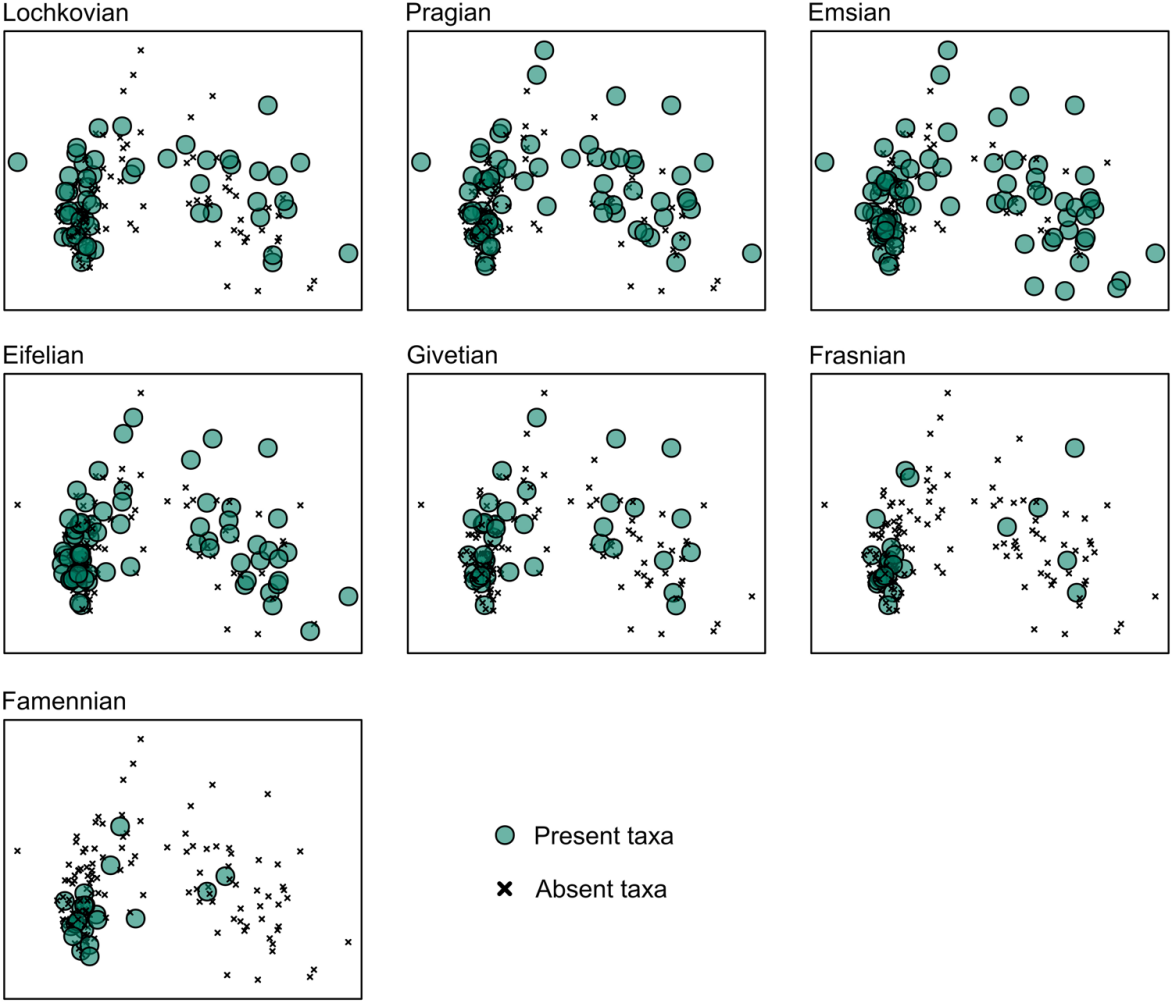
Morphospace for Lochkovian - Famennian trilobites using data from TriloMorph. Accentuated data points indicate morphotypes present in the respective intervals, morphotypes that are absent (x) are also indicated in order to show the total spectrum.

In our example, the Devonian data from TriloMorph combined with the PBDB shows correspondence between taxonomic and morphological diversity during the Lower and Middle Devonian, where the highest disparity values are related to moments of high taxonomic diversity (Fig. 9). However, towards the Eifelian–Givetian diversity decreases significantly in relation to disparity, with a clear decoupling during the Frasnian. These trends rule out random extinctions as disparity does not remain stable, rather it slowly decreases towards the Upper Devonian. This, in relation to morphospace occupation patterns (Fig. 10), suggest selective extinctions towards the margin, characterised by moderate reductions of the SoV paired with asymmetric reductions of datapoints^112^.

For large scale analyses, the dataset is meant to be used in conjunction with occurrence data (obtained from the PBDB for example). Indeed, morphological information from TriloMorph and occurrence and higher taxonomic information from the PBDB can be merged according to the desired taxonomic resolution using the genus or species names (Fig. 1: Data recollection). The ‘TriloMorph-workflow’ R script associated with the database illustrates in detail how to merge together occurrence data (such as from the PBDB) to the TriloMorph geometric data and to analyse them to produce disparity curves for example (see Figs. 4, 5, 9, 10).

### Supplementary information

TriloMorph R functions

TriloMorph workflor in R

## Data Availability

The R script and functions written by the authors used in the analysis are available at the following GitHub repository: https://github.com/balsedie/trilomorph. Analyses have been computed with R version 4.1.3^113^ with specific functions (available at Github) and the package geomorph version 4.0.1^57,58^.
